# Abnormal hubs in global network as potential neuroimaging marker in generalized anxiety disorder at rest

**DOI:** 10.3389/fpsyg.2022.1075636

**Published:** 2022-12-16

**Authors:** Lili Meng, Yuandong Zhang, Hang Lin, Jingping Mu, Heng Liao, Runlan Wang, Shufen Jiao, Zilong Ma, Zhuangzhuang Miao, Wei Jiang, Xi Wang

**Affiliations:** ^1^Department of Psychiatry, Wuhan Mental Health Center, Wuhan, China; ^2^Department of Sleep, Wuhan Hospital for Psychotherapy, Wuhan, China; ^3^Clinical College, Wuhan University of Science and Technology, Wuhan, China; ^4^Department of Mental Health, Taihe Hospital, Hubei University of Medicine, Shiyan, China; ^5^Department of Neurosurgery, Tongji Hospital, Tongji Medical College, Huazhong University of Science and Technology, Wuhan, China

**Keywords:** generalized anxiety disorder, degree centrality, magnetic resonance imaging, receiver operating characteristic, middle temporal gyrus

## Abstract

**Background:**

Mounting studies have reported altered neuroimaging features in generalized anxiety disorder (GAD). However, little is known about changes in degree centrality (DC) as an effective diagnostic method for GAD. Therefore, we aimed to explore the abnormality of DCs and whether these features can be used in the diagnosis of GAD.

**Methods:**

Forty-one GAD patients and 45 healthy controls participated in the study. Imaging data were analyzed using DC and receiver operating characteristic (ROC) methods.

**Results:**

Compared with the control group, increased DC values in bilateral cerebellum and left middle temporal gyrus (MTG), and decreased DC values in the left medial frontal orbital gyrus (MFOG), fusiform gyrus (FG), and bilateral posterior cingulate cortex (PCC). The ROC results showed that the DC value of the left MTG could serve as a potential neuroimaging marker with high sensitivity and specificity for distinguishing patients from healthy controls.

**Conclusion:**

Our findings demonstrate that abnormal DCs in the left MTG can be observed in GAD, highlighting the importance of GAD pathophysiology.

## Introduction

Generalized anxiety disorder (GAD) is characterized by chronic, diffuse, unrealistic state anxiety about some life situations and often manifested as persistent mental stress accompanied by dizziness, chest tightness, palpitations and other physical symptoms, which is the most common anxiety disorder, with high incidence rate, prolonged course, recurrent attacks, high comorbidity rate with physical diseases and other mental diseases, and heavy disease burden (Richards et al., 2015; Stein and Sareen, 2015). GAD is currently lack of effective objective biological markers, which are mainly diagnosed through symptomatology, because it overlaps with other anxiety and depression disorders in symptomatology (Blazer et al., 1991; Grados et al., 2005). In clinical work, based on symptomatic diagnosis and prognosis evaluation, misdiagnosis often occurs. Therefore, it is very important to explore potential neuroimaging markers for the diagnosis and prognosis of GAD.

Functional neuroimaging technology has been widely used in the study of neuropsychiatric diseases (Gao et al., 2021, 2022a,b,c; Lin et al., 2022) including GAD because of its excellent temporal and spatial resolution, as well as its medium advantages of safety, non-invasive and no exposure to radioactive substances (Logothetis et al., 2001). A large number of functional magnetic resonance studies have found that GAD patients have abnormal changes in multiple related neural networks in multiple functional abnormal brain regions, which indicates that the pathogenesis of GAD may be related to the abnormal synthesis of functional networks in multiple brain regions, rather than the function of a single brain region caused by an abnormality in the network (Bashford-Largo et al., 2022; Monteiro et al., 2022). Previous studies have found that the functional connectivity of the amygdala and prefrontal cortex in GAD patients is decreased, and some studies have found that local signals in these brain regions are decreased, such as local consistency and low-frequency amplitude (De Bellis et al., 2000). However, there are also some studies with opposite results. It is worth noting that the abnormal brain regions in GAD patients are often not bilaterally symmetrical, and the activation of frontal lobes in different hemispheres is associated with different symptoms of GAD (Yassa et al., 2012). At least the following two factors can explain this difference: First, patients with anxiety disorders subtypes are not screened, and it is difficult to guarantee the homogeneity of patients; second, the research methods and the analysis software used are inconsistent (Gray et al., 2019; Costache et al., 2020). Therefore, it is necessary to choose GAD with high homogeneity as the research object to explore the characteristics of brain functional network in GAD patients, clarify the brain imaging mechanism of GAD, and find the methods of early diagnosis and treatment of GAD.

Degree centrality (DC), recently been applied to reveal the core of brain networks. An increase in voxel-wise DCs in brain regions indicates an increased degree of their global connectivity, whereas a decrease in voxel-wise DCs in brain regions indicates a decreased degree of their global connectivity. Previous researchers have used DC to study different neuropsychiatric diseases such as major depressive disorder (Guo et al., 2022), attention deficit and hyperactivity disorder (Zhou et al., 2019), bipolar disorder (Deng et al., 2019), and schizophrenia (Yu X. M. et al., 2021). However, the changes of brain DC values in GAD patients remain unclear.

In the present study, we hypothesized that abnormal DCs were associated with clinical variables in GAD patients and could serve as potential neuroimaging markers to differentiate GAD from healthy controls.

## Materials and methods

### Participants

Thank you very much for your suggestions. We have sorted out the inclusion criteria and exclusion criteria of subjects in the manuscript submitted this time.

41 GAD patients were recruited from Taihe Hospital affiliated to Hubei Medical University. Inclusion criteria are as follows: (1) Conform to GAD diagnostic criteria in Diagnostic and Statistical Manual of Mental Diseases (DSM-5); (2) Age 18–55, normal intelligence; (3) Han nationality, right hand; (4) Hamilton Anxiety Scale (HAMA) score ≥ 24.

The exclusion criteria were as follows: (1) Current or past medical history of mental disorders other than GAD diagnosed by DSM-5; (2) History of mental or neurological diseases of first-degree relatives; (3) Previous history of brain trauma, alcohol or drug dependence; (4) Have used any psychotropic drugs within 24 h (5) Breastfeeding and pregnant women; (6) Patients with contraindications to MRI examination.

Forty-five healthy people with gender, age and education level matching GAD group were recruited as healthy control (HC) through advertisement.

Inclusion criteria: (1) HAMA score <8; (2) Han nationality, right hand; (3) No history of neuropsychiatric disease;

Exclusion criteria are the same as those of GAD patients.

Our research was recognized by the Ethics Committee of Taihe Hospital affiliated to Hubei Medical University, and all participants signed a written informed consent form.

### MRI acquisition and processing

Resting-state fMRI data were obtained using a 3.0 T Philips MRI at Taihe hospital. All subjects were asked to stay awake with their eyes closed. Resting-state functional images were acquired using echo-planar imaging sequences with the following parameters: repetition time/echo time (TR/TE) 2000/30 ms, 31 slices, 220 × 220 matrix, 90° flip angle, 24 cm field of view, 5 mm thick layers without gaps. Functional MRI data processing was performed using the data processing assistant for rs-fMRI (Chao-Gan and Yu-Feng, 2010), which works with SPM12 implemented in MATLAB. converts the DICOM format images obtained by scanning into NIFTI format, and removes the images at the first 10 time points. Taking into account the time when the machine magnetic field reaches stability and the adaptation time of the subject to the environment. Image preprocessing is performed on the converted data. The processing process includes: slice timing due to the different time obtained at each layer; Estimating head motion parameters during scanning and performing head motion correction; The head movement corrected image space was normalized to the Montreal Neurological Institute (MNI) standard space, and the voxel size was resampled to 3 mm × 3 mm × 3 m (Normalization). The image is subjected to de linear drift and filter (0.01–0.08 Hz) to reduce low-frequency drift and filter high-frequency physiological noise (such as breathing and heartbeat). The next step is to analyze the subjects whose translational motion in each direction (*x*, *y*, *z*) is <2 mm and whose rotational angle is <2°.

### DC calculation

DC values were calculated using “Rest-DC” toolkit in rest package.^1^ The Pearson correlation coefficient is used to calculate the DC measure, and the Pearson correlation coefficient between all voxel pairs of time series is used to construct the graph of each participant. In this figure, each brain voxel is regarded as a node, and the significant correlation between nodes is regarded as an edge, and then the n * n matrix of Pearson correlation coefficient between each pair of voxels is obtained to establish each subjective whole brain functional connection. Next, in order to improve the normality, Fisher’s r-to-z transform is applied to transform each correlation matrix into a Z-score matrix. The sum of the Z values between the voxels and the selected voxels and all other voxels is then calculated to generate a weighted DC for the voxels. In addition, in order to eliminate the potential false connectivity, the threshold of Pearson correlation coefficient was set at *r* > 0.25 (Zhou et al., 2021) by thresholding each correlation at *p* < 0.01.

### Statistical analysis

All statistical analyses were performed using SPSS 23.0 (SPSS Inc., Chicago, IL, United States). Independent two-sample *t*-tests and chi-square tests were used to determine demographic differences between GAD patients and HCs.

### Correlation analysis

DC values were extracted from abnormal brain regions between the two groups. To explore the difference of DC between MDD patients and HCs, a voxel-by-voxel two-sample *t*-test was performed. The significance threshold was set at *p* < 0.01. The abovementioned *t*-tests were performed with gender, age, and years of education as covariates as these factors may confound the results. Pearson correlation coefficients were calculated to detect correlations between abnormal DC values and clinical variables.

## Results

### Demographic and clinical variables

Demographic and clinical variables are shown in Table 1. There was no significant difference between GAD patients and HCs in terms of gender-, age- and educational level (*p* > 0.01).

**Table 1:** Demographic information.

Characteristics	Patients (*n* = 41)	Controls (*n* = 45)	*χ*^2^ or *T*	Value of *p*
Gender (male/female)	41 (16/25)	45 (18/27)	3.77	0.05^a^
Age (years)	35.09 ± 2.54	35.20 ± 3.50	3.14	0.08^b^
Education (years)	9.12 ± 2.68	10.56 ± 2.03	1.41	0.24^b^
HAMA (scores)	27.90 ± 2.14			

HAMA, Hamilton Anxiety Scale.

a The *p*-value for gender distribution was obtained by chi-square test.

b The *p*-value were obtained by two sample *t*-tests.

### Significant DC difference across groups

**Figure 1:**
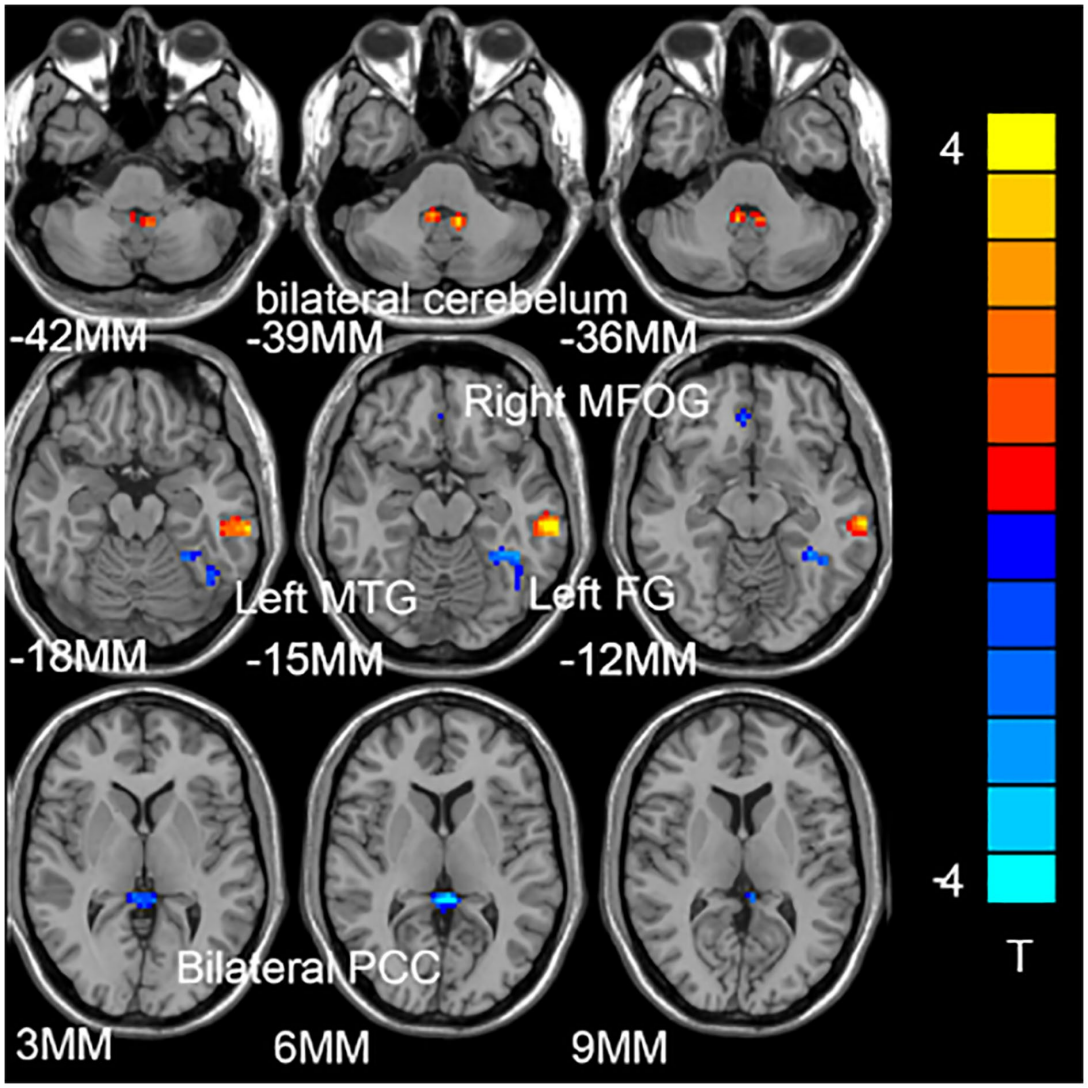
Compared with the healthy control subjects (HCs), the brain regions of patients with generalized anxiety disorder (GAD) have abnormal degree centrality (DC) values. The blue represents the decrease in DC value, and the red represents the increase in DC value.

Figure 1 shows the significant difference in DC values between the two groups (GAD and HCs). Compared with the control group, increased DC values in bilateral cerebellum and left middle temporal gyrus (MTG), and decreased DC values in the left medial frontal orbital gyrus (MFOG), fusiform gyrus (FG), and bilateral posterior cingulate cortex (PCC; Figure 1; Table 2).

**Table 2:** Alterations of DC between patients and controls. (at baseline, after treatment) and controls.

Cluster location	Peak (MNI)			Number of voxels	*T*-value
	*X*	*Y*	*Z*		
Bilateral cerebellum	±9	−48	−39	46	3.71
Left middle temporal gyrus	6	−48	18	111	4.03
Left fusiform gyrus	51	42	6	30	−3.38
Right medial orbital frontal gyrus	9	63	24	67	−3.51
Bilateral posterior cingulate cortex	±39	−45	45	30	−3.86

DC, degree centrality; MNI, Montreal Neurological Institute.

### ROC of DC value analysis

**Figure 2:**
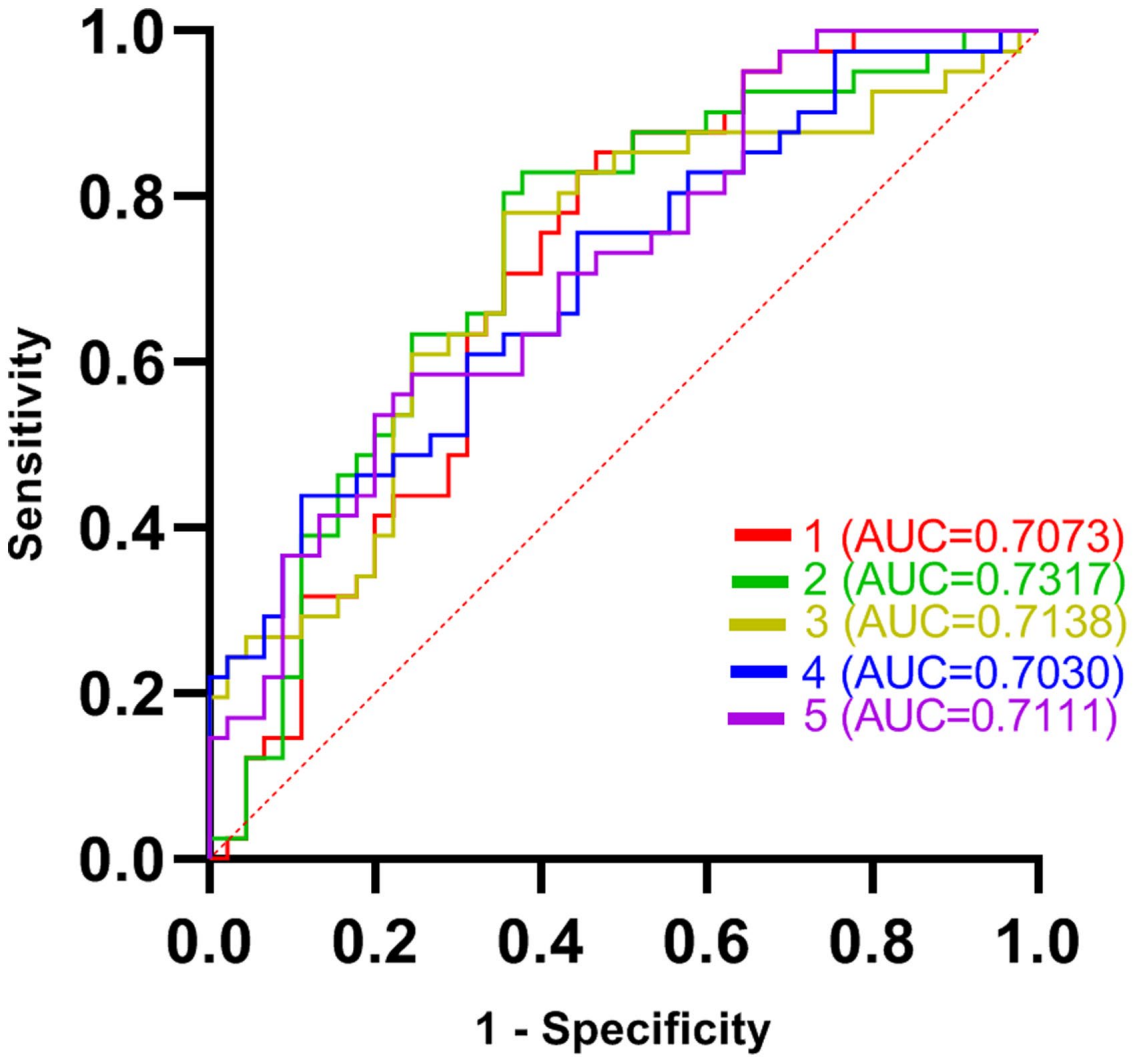
The receiver operating characteristic (ROC) curves of brain regions with abnormal degree centrality (DC) values in discriminating generalized anxiety disorder (GAD) patients from healthy controls (HCs). AUC represents the area enclosed by the coordinate axis under the ROC curve. Curve 1 represents the bilateral cerebellum; Curve 2 represents the left middle temporal gyrus; Curve 3 represents the left fusiform gyrus; Curve 4 represents the right medial orbital frontal gyrus; Curve 5 represents the bilateral posterior cingulate cortex.

**Figure 3:**
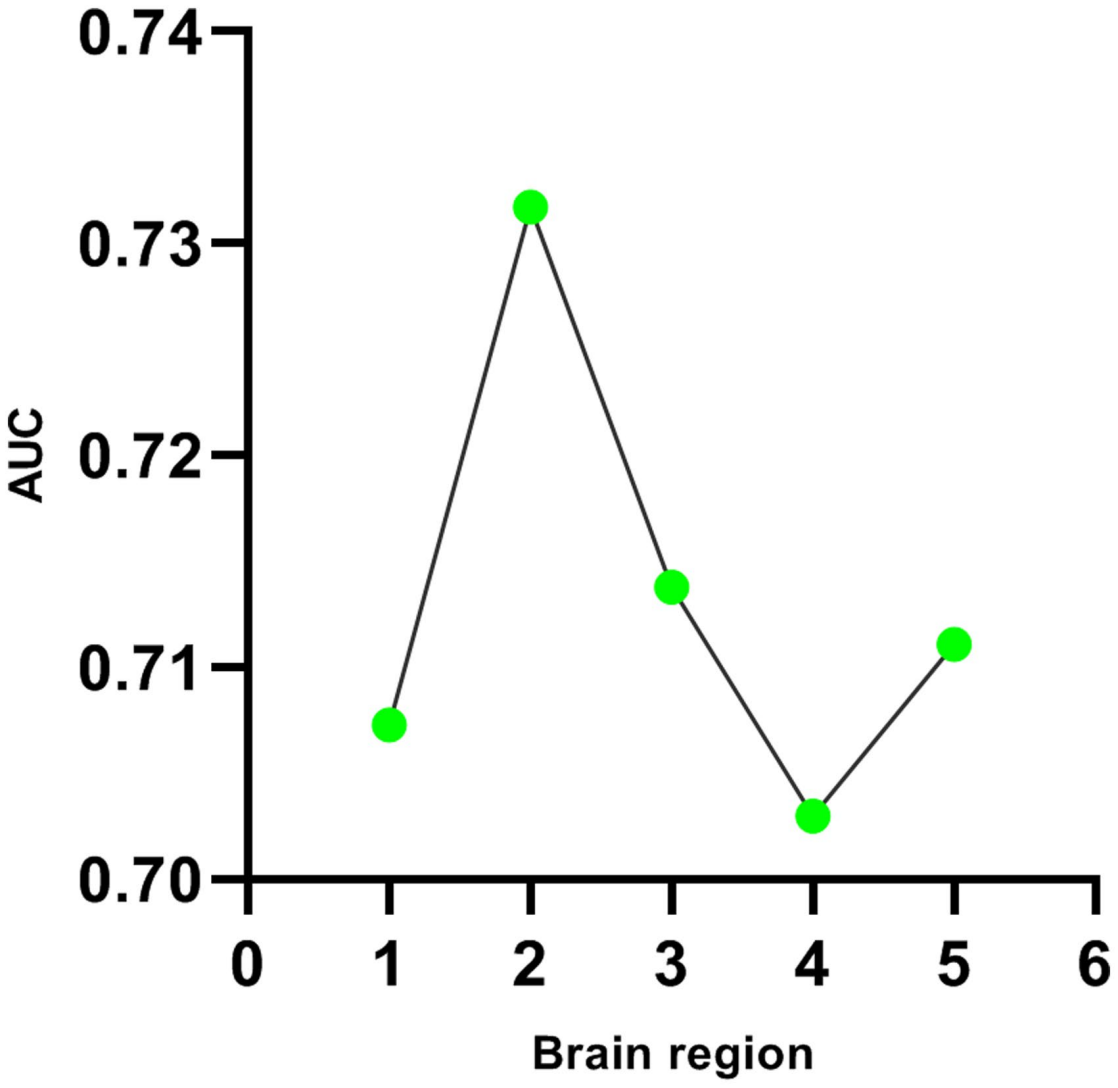
Line diagram of AUC in brain regions with abnormal DC values. The first green dot represents the bilateral cerebellum; The second green dot represents the left middle temporal gyrus; The third green dot represents the left fusiform gyrus; The fourth green dot represents the right medial orbital frontal gyrus; The fifth green dot represents the bilateral posterior cingulate cortex.

Five DC abnormal regions (bilateral cerebellum, left MTG, left MFOG, FG, and bilateral PCC) were observed in the patient group. Further analysis of this result revealed that the abnormal DC value in the left PCC showed the highest AUC (0.7317; Figures 2, 3).

### Correlations between DC values and clinical variables

There was no significant correlation between DC values and clinical data.

## Discussion

As a core index to measure network performance, degree centrality has been applied to the research of brain disease network in recent years, which has helped to explore the mechanism of brain network in brain disease imaging and the diagnosis and prognosis of brain functional diseases (Guo et al., 2022). Forty-one GAD patients and 45 health controls participated in the study. We found that there were increased DC values in bilateral cerebellum and left middle temporal gyrus (MTG), and decreased DC values in the left medial frontal orbital gyrus (MFOG), fusiform gyrus (FG), and bilateral posterior cingulate cortex (PCC) comparing to health controls. Furthermore, ROC has been used for biomedical applications in the diagnosis of GAD, and found that the DC value of the left MTG successfully discriminate the two groups with highest degree of accuracy and AUC (0.7317).

The temporal lobe, including the parahippocampal gyrus, FG, amygdala, and entorhinal cortex (Kaur et al., 2022), is often the target of functional and structural studies of GAD (De Bellis et al., 2000). MTG plays a key role in language, emotion and long-time memory (Preston and Wagner, 2007). Neuroimaging has also repeatedly revealed activation of abnormal regions of the MTG, which act as third visual associative brain regions and are associated with cognitive functions such as memory, language, and visual perception (Binney et al., 2010). Consistent findings from neuroimaging studies suggest that the temporal lobe is involved in emotional processing and social cognition (Uno et al., 2010). Furthermore, the MTG is a key node in a broad network of frontal, parietal, occipital, and subcortical structures. Therefore, this abnormally activated region of MTG may also affect the function of the temporal lobe. In this study, our results suggest that increased DCs in MTG may be associated with emotional and cognitive deficits in GAD patients, although cognitive tests were not assessed in this study. In addition, the DC value in the right MTG of the patient group was decreased, which may explain the clinical manifestations of cognitive decline such as learning, memory, and attention in GAD patients. However, no correlation was found between symptom severity and disease duration in patients with GAD and the reduction in DC values, suggesting that the reduction in DC values is a characteristic change in GAD independent of the severity of symptoms in patients. Furthermore, abnormal DC values of MTG can strongly differentiate patients from controls. The ROC results also support the notion that reduced DC values in the MTG may be a characteristic change in GAD.

In this study, reduced DC in the FG was revealed in GAD relative to healthy controls, which implies dysfunction in these regions might be associated with the pathogenesis for GAD. FG locates in the visual recognition network, a part of the temporal lobe, is the facial recognition area of human brain, which is involved in negative cognitive model. T Furmark et al. revealed that decreased reactivity in the bilateral fusiform gyrus in response to fearful faces, as well as increased connectivity between the fusiform gyrus and amygdala, and decreased connectivity between the fusiform gyrus and ventromedial prefrontal cortex in GAD (Mansson et al., 2013). Decreased fusiform connectivity during processing of fearful faces in social anxiety disorder (Cui et al., 2020a). From the perspective of dynamic local brain activity, recent two studies found that GAD exhibited increased dynamic ALFF variability in widespread regions, including the bilateral FG, dorsomedial prefrontal cortex, orbital frontal gyrus, inferior parietal lobule, and temporal gyrus (Chen et al., 2020b; Cui et al., 2020b). In addition, A Meta-Analysis based on fMRI studies showed patients with GAD had significantly lower activation of the left cerebellar and FG (Yu X. et al., 2021). These findings might be associated with the facial mood perception and negative cognition, and it may be the part of the neuropathological mechanism of social withdrawal symptoms in GAD.

The frontal lobe is involved in cognitive control. The main function of the prefrontal lobe is to participate in high-level cognitive functions, including attention, thinking and task execution (Mcallister-Williams et al., 2010). Previous MRI studies on anxiety neural circuits found that the activation of the prefrontal lobe was enhanced in the process of emotional activation (Etkin et al., 2009). It also plays a compensatory role and regulate fear related circuits such as amygdala and insula. Abnormal structural and functional alterations in the frontal lobe have been also reported in many previous reports in patients with GAD (Chen et al., 2020a; Porta-Casteras et al., 2020). For example, Schienle et al. found that the volume of the dorsal prefrontal cortex of GAD patients increased by structural phase scanning, which may be the compensatory increase caused by the pathological high activation of anxiety stimulation in this brain region of GAD patients (Schienle et al., 2011). Etkin et al. found that the functional connection between bilateral amygdala and dorsal prefrontal lobe was enhanced in GAD patients at rest, suggesting that the functional connection between prefrontal lobe and other brain regions also participated in the compensatory effect (Etkin et al., 2009). In this study, we found that the DC value of the MFOG in GAD patients was lower than that in the control group suggesting that the MFOG of GAD patients also had abnormal functions.

PCC, the master node of default mode network (DMN), is functionally involved in visual spatial information, mental image, metaphor understanding and episodic memory (Greicius et al., 2003). Previous studies have shown that PCC is involved in the pathogenesis of GAD (Porta-Casteras et al., 2020). Regarding its structure, researchers have demonstrated that there is a positive correlation between the gray matter volume of PCC and conceptual creativity. In addition, a large number of studies have shown that the enhancement of the cortical surface area of PCC mainly contributes to the morphological changes of the sagittal brain in adults. In addition, recent studies have shown that PCC may also play an important role in mental imagery and the correlation between vivid memory and egocentric perspective, and its enhanced capacity is related to the tendency to recall autobiographical memories of egocentric plots (Yang et al., 2022). High language innovation ability, including high fluency, originality and flexibility, indicates that the regional functional homogeneity of PCC is reduced. These symptoms are also the most common symptoms in GAD patients. The meta-analysis showed that PCC is a driving recognition activated by familiarity (Busler et al., 2019). PCC also promotes consciousness networks in the nervous system, and GAD patients have selective hypometabolism. In addition, Uretone et al. have found that adolescents with anxiety disorder are abnormally activated in the task state. This study also found that the DC value of bilateral PCC in GAD patients was lower than that of normal controls, suggesting that the patients may be in abnormal emotional integration at rest, leading to anxiety and other symptoms that cannot be controlled by clinical manifestations.

This study has several limitations. First, the sample size of this study is small. Second, all study subjects focused on middle-aged people and could not cover the characteristics of GAD patients in other age groups. Third, we did not recruit patients with various types of anxiety disorders. Therefore, it is difficult to further explore the neurobiological differences of different subtypes of GAD.

Taken together, our findings in this study suggest that GAD patients have a unique DC pattern. Decreased DC values of the left MTG may be a stable and unique neurobiological feature of GAD.

## Data availability statement

The raw data supporting the conclusions of this article will be made available by the authors, without undue reservation.

## Ethics statement

The studies involving human participants were reviewed and approved by the medical ethics committee of Taihe Hospital affiliated to Hubei University of Medicine. The patients/participants provided their written informed consent to participate in this study. Written informed consent was obtained from the individual(s) for the publication of any potentially identifiable images or data included in this article.

## Author contributions

JM, HeL, RW, SJ, and ZiM conceived the project idea. LM, YZ, and HaL implemented the method and performed the experiments. ZhM supervised the project. WJ provided critical suggestions for the design of the experiment. XW directed the revision of the paper. All authors contributed to the article and approved the submitted version.

## Funding

The investigation was supported by the grant from the Guided Scientific Research Project of Shiyan City (no. 21Y21) and Wuhan Young and Middle aged Medical Backbone Talents Fund (2021ZQNYX002).

## Conflict of interest

The authors declare that the research was conducted in the absence of any commercial or financial relationships that could be construed as a potential conflict of interest.

## Publisher’s note

All claims expressed in this article are solely those of the authors and do not necessarily represent those of their affiliated organizations, or those of the publisher, the editors and the reviewers. Any product that may be evaluated in this article, or claim that may be made by its manufacturer, is not guaranteed or endorsed by the publisher.

## Footnotes

^1^ http://www.restfmri.net/
